# Dysbiotic gut fungi exacerbate *Klebsiella pneumoniae* lung infection via Dectin-1-mediated alveolar macrophage hyperactivation

**DOI:** 10.1093/ismejo/wraf181

**Published:** 2025-08-16

**Authors:** Shengfu He, Yating Sun, Jiawen Yu, Mingyang Tang, Qingyue Zhang, Bao Meng, Renyu Fan, Zhiqiang Liu, Yanyan Liu, Lifen Hu, Ting Wu, Jiabin Li

**Affiliations:** Department of Gastroenterology, The First Affiliated Hospital of Anhui Medical University, Hefei, Anhui 230022, China; Department of Infectious Diseases & Anhui Center for Surveillance of Bacterial Resistance, The First Affiliated Hospital of Anhui Medical University, Hefei, Anhui 230022, China; Anhui Province Key Laboratory of Infectious Diseases & Institute of Bacterial Resistance & Institute of Infectious Diseases, Anhui Medical University, Hefei, Anhui 230022, China; Department of Infectious Diseases & Anhui Center for Surveillance of Bacterial Resistance, The First Affiliated Hospital of Anhui Medical University, Hefei, Anhui 230022, China; Anhui Province Key Laboratory of Infectious Diseases & Institute of Bacterial Resistance & Institute of Infectious Diseases, Anhui Medical University, Hefei, Anhui 230022, China; Health Management Center, The First Affiliated Hospital of Anhui Medical University, Hefei, Anhui 230022, China; Department of Oncology, Anqing First People’s Hospital of Anhui Medical University/Anqing First People’s Hospital of Anhui Province, Anqing, Anhui 246052, China; Anhui Province Key Laboratory of Infectious Diseases & Institute of Bacterial Resistance & Institute of Infectious Diseases, Anhui Medical University, Hefei, Anhui 230022, China; Health Management Center, The First Affiliated Hospital of Anhui Medical University, Hefei, Anhui 230022, China; Anhui Province Key Laboratory of Infectious Diseases & Institute of Bacterial Resistance & Institute of Infectious Diseases, Anhui Medical University, Hefei, Anhui 230022, China; Health Management Center, The First Affiliated Hospital of Anhui Medical University, Hefei, Anhui 230022, China; Department of Infectious Diseases & Anhui Center for Surveillance of Bacterial Resistance, The First Affiliated Hospital of Anhui Medical University, Hefei, Anhui 230022, China; Anhui Province Key Laboratory of Infectious Diseases & Institute of Bacterial Resistance & Institute of Infectious Diseases, Anhui Medical University, Hefei, Anhui 230022, China; Department of Gastroenterology, The First Affiliated Hospital of Anhui Medical University, Hefei, Anhui 230022, China; Department of Gastroenterology, The First Affiliated Hospital of Anhui Medical University, Hefei, Anhui 230022, China; Department of Infectious Diseases & Anhui Center for Surveillance of Bacterial Resistance, The First Affiliated Hospital of Anhui Medical University, Hefei, Anhui 230022, China; Anhui Province Key Laboratory of Infectious Diseases & Institute of Bacterial Resistance & Institute of Infectious Diseases, Anhui Medical University, Hefei, Anhui 230022, China; Department of Infectious Diseases & Anhui Center for Surveillance of Bacterial Resistance, The First Affiliated Hospital of Anhui Medical University, Hefei, Anhui 230022, China; Anhui Province Key Laboratory of Infectious Diseases & Institute of Bacterial Resistance & Institute of Infectious Diseases, Anhui Medical University, Hefei, Anhui 230022, China; Department of Infectious Diseases & Anhui Center for Surveillance of Bacterial Resistance, The First Affiliated Hospital of Anhui Medical University, Hefei, Anhui 230022, China; Anhui Province Key Laboratory of Infectious Diseases & Institute of Bacterial Resistance & Institute of Infectious Diseases, Anhui Medical University, Hefei, Anhui 230022, China; Department of Infectious Diseases & Anhui Center for Surveillance of Bacterial Resistance, The First Affiliated Hospital of Anhui Medical University, Hefei, Anhui 230022, China; Anhui Province Key Laboratory of Infectious Diseases & Institute of Bacterial Resistance & Institute of Infectious Diseases, Anhui Medical University, Hefei, Anhui 230022, China

**Keywords:** gut fungi, *Klebsiella pneumoniae;* dectin-1, alveolar macrophage, gut-lung axis

## Abstract

Escalating antibiotic resistance of *Klebsiella pneumoniae* underscores the urgent need for therapeutic strategies. Whereas gut bacterial dysbiosis exacerbates pulmonary infections, the role of gut fungi in modulating lung immunity remains understudied. Here, we demonstrate that antibiotic-induced gut fungal expansion aggravates pneumonia by enhancing alveolar macrophage-driven inflammation via Dectin-1 signaling. Clinical analyses demonstrated that pneumonia patients receiving ineffective prehospital antibiotic therapy showed gut bacterial depletion accompanied by fungal overgrowth (primarily *Candida* spp.), with a positive correlation observed between fungal abundance and hospitalization duration. In murine models, antibiotic-induced gut microbiota disruption promoted fungal proliferation, subsequently upregulating Dectin-1 expression in alveolar macrophages. This activation triggered excessive IL-1β secretion and neutrophil recruitment, exacerbating lung injury and mortality. Our results demonstrated that both antifungal intervention and Dectin-1 knockout reversed these pathological effects, resulting in improved survival rates, reduced bacterial dissemination, and attenuated inflammatory cytokine levels. Mechanistically, gut fungi remotely potentiated pulmonary inflammation through the alveolar macrophage “Dectin-1/IL-1β/neutrophil axis”, independent of pathogen clearance. Although recent studies have begun to uncover “mycobiome-lung” disease associations, our findings specifically demonstrate that fungal dysbiosis mediates the “gut-lung axis” during multidrug-resistant *K. pneumoniae* infections. This study provides mechanistic insights into microbial crosstalk and advances translational approaches for combating antibiotic-exacerbated pneumonias.

## Introduction

*Klebsiella pneumoniae* is a globally prevalent pathogen capable of causing life-threatening diseases [1]. The antibiotic resistance of *K. pneumoniae* is increasing due to the overuse of antibiotics and treatment options for *K. pneumoniae* infections are limited due to the emergence of multidrug-resistant strains [2]. Therefore, there is an urgent need to explore novel therapeutic strategies. With the continuous development of high-throughput sequencing technology, the crucial role of the gut microbiota in the body’s defense against external pathogens is increasingly being revealed. Among these, the regulation of the gut microbiota on the lung’s immune response has been widely studied [3, 4]. Antibiotic-induced gut bacterial dysbiosis has been shown to cause pulmonary immune dysregulation, impairing clearance of *Streptococcus Pneumoniae* infections [5]. Previous studies have demonstrated that vancomycin-colistin administration induces gut bacterial dysbiosis, which strongly impairs monocyte/dendritic progenitors and lung immunity, thereby worsening the outcomes of *Pseudomonas aeruginosa* lung infection [6]. Additionally, antibiotic usage impairs both innate and adaptive antiviral immune responses in mice, which substantially increases the severity of viral respiratory infections such as influenza [7] and severe acute respiratory syndrome coronavirus 2 (SARS-CoV-2) [8]. A study found that antibiotic use in early life disrupts the gut bacterial community and exacerbates house dust mite (HDM)-induced allergic airway inflammation, thereby increasing the risk of developing allergies and asthma [9]. Although recent research efforts have focused on understanding how gut microorganisms influence respiratory health, most of these studies have concentrated on the role of gut bacteria and ignored the influence of other gut microorganisms.

Gut bacteria and archaea microbiota consist of trillions of commensal microorganisms, which have been shown to influence multiple aspects of human physiology and disease [10]. However, the gut microbiota also includes representatives from other kingdoms, such as fungi, viruses, and parasites [11]. Although fungi account for only 0.01%–2% of the gut microbiota, their significant impact on health and disease is sufficient to offset their numerical disadvantage [12]. Under homeostatic conditions in healthy individuals, commensal bacteria and fungi maintain a relatively balanced state through competitive inhibition and metabolic cross-feeding. Normal commensal bacteria in the gut provide an important defense mechanism against the overcolonization of commensal fungi [13]. When normal gut bacteria are disrupted (e.g. by antibiotic usage), it can lead to gut fungal overgrowth [14, 15]. Our previous study has shown that depleting gut bacteria by antibiotic cocktail (Abx) therapy in mice can exacerbate symptoms of *K. pneumoniae* pneumonia [16, 17]. This inevitably leads us to propose new hypotheses regarding the changes that occur in gut fungi during the Abx treatment. Whether altered gut fungi contribute to the aggravated *K. pneumoniae* pneumonia in Abx-treated mice remains unclear. In addition, gut fungi have recently been recognized as regulators of inflammatory responses in many diseases, such as various types of cancers, infectious diseases, and autoimmune diseases [18–20]. Thus, we hypothesized that antibiotic-induced bacterial depletion, a well-documented cause of gut fungal dysbiosis, drives the exacerbation of *K. pneumoniae* pneumonia through the gut-lung axis.

Dectin-1, as a member of the C-type lectin receptor family, is a key innate immune receptor for sensing fungi, primarily expressed on activated myeloid cells such as macrophages, dendritic cells, and neutrophils [21]. Previous studies have shown that gut fungi regulate intestinal immunity by activating Dectin-1 receptors [22]. Gut fungi can also remotely regulate the expression of Dectin-1 in tissues and organs outside the intestine [15]. The “gut-liver axis” research reveals that gut fungi stimulate Dectin-1 receptors on Kupffer cells, triggering IL-1β release that exacerbates hepatocyte damage [23]. Emerging evidence demonstrates that Dectin-1 plays a critical role in cardiac pathophysiology. Following myocardial ischemia/reperfusion injury, macrophage Dectin-1 upregulation enhances IL-1β secretion and neutrophil recruitment at the injury site [24]. Mechanistically, genetic ablation of Dectin-1 attenuates cardiac remodeling and dysfunction by suppressing neutrophil infiltration and inflammatory cytokine production [21]. An unresolved question is whether gut fungi contribute to Dectin-1-mediated cardiac protection, because this aspect was not addressed in previous research. In this study, we demonstrate that the use of antibiotics depletes gut bacteria and leads to the disruption and expansion of gut fungi. We found that the depletion of normal gut bacteria exacerbates *K. pneumoniae* pneumonia in Abx-treated mice. In contrast, the expanded gut fungi also worsen the symptoms of *K. pneumoniae* pneumonia in Abx-treated mice by upregulating the Dectin-1 receptor in mouse alveolar macrophages (AMs), resulting in a series of inflammatory changes in the mouse lungs. Together, our study demonstrates that gut fungi shape the lung’s anti*–K. pneumoniae* immunity.

## Materials and methods

Detailed methods can be found in the supplementary materials.

### Subject recruitment and fecal sample collection

17 hospitalized patients with community-acquired pneumonia (CAP) were enrolled in this study. 10 patients who received oral antibiotics prior to admission yet showed treatment failure, defined as: (i) persistent fever (≥38°C) after 72 h of therapy; (ii) WBC count >10 × 10^9^/L at admission; (iii) thoracic CT scans demonstrated features indicative of pulmonary inflammatory changes (antibiotics group). The other seven had not received oral antibiotics before admission (control group). All participants were of East Asian descent, ethnic Han, and yellow race. Fecal samples from all patients were collected prior to the start of antibiotic therapy during hospitalization. Detailed information ~17 participants who provided fecal specimens was recorded (Table S1). After collection, fecal samples were transported to the laboratory using liquid nitrogen and stored at −80°C until analysis. The study protocol was approved by the Committee on Medical Ethics of the First Affiliated Hospital of Anhui Medical University (approved no. PJ2024-08-42). All procedures were performed in accordance with approved guidelines. Informed consent was obtained from all patients, who were fully informed about the study.

### Mice

The *Clec7a* knockout mouse model used in this study was designed and developed by the Shanghai Model Organisms Center, Inc. (Shanghai, China). The generation of *Clec7a* mice is described in the supplementary material. Wild-type (WT) C57BL6/J mice (weight, 20–25 g; male; 8–10 weeks old) were purchased from GemPharmatech Co., Ltd. All mice were housed in a standard specific-pathogen-free (SPF) environment. For Abx treatment, mice were administered broad-spectrum antibiotics (ampicillin, 1 g/l; neomycin sulfate, 1 g/l; metronidazole, 1 g/l; and vancomycin, 0.5 g/l) in drinking water for 14 days [16]. For Abx + Antifungal (AF) treatment, mice were treated with Abx for 14 days and on the seventh day, 0.5 g/l fluconazole was added to the drinking water [15]. Mice were anesthetized with isoflurane (5%) followed by cervical dislocation, with death verification via absence of vital signs. The animal study was reviewed and approved by the Animal Experimentation Ethics Committee of Anhui Medical University (approval no. LLSC20190253) and experiments were performed in strict accordance with the Animal Research: Reporting of *in vivo* Experiments (ARRIVE) guidelines for the care and use of laboratory animals.

### Bacterial strains and *K. Pneumoniae* infection

*Klebsiella pneumoniae* (ATCC 43816) was cultured in Luria broth (LB) at 37°C overnight. Abx and Abx + AF treatments were discontinued 3 days prior to infection. Mice were then sufficiently anesthetized with pentobarbital sodium and inoculated intranasally with 50 μL of sterile phosphate-buffered saline (PBS) containing 1 × 10^5^ CFU of *K. pneumoniae.*

### Microbiota analysis

Fecal samples from experimental mice were collected prior to euthanasia and stored at −80°C. Total genomic DNA was isolated using MagPure Soil DNA LQ Kit (Magan) following the manufacturer’s instructions. DNA concentration and integrity were measured with NanoDrop 2000 (Thermo Fisher Scientific, USA) and agarose gel electrophoresis. The extracted DNA was used as template for PCR amplification of bacterial 16S rRNA genes with the barcoded primers and Takara Ex Taq (Takara).

To analyze bacterial diversity, the V3-V4 hypervariable regions of the 16S rRNA genes were targeted and amplified using universal primers 343 F: TACGGRAGGCAGCAG; 798 R: AGGGTATCTAATCCT. For fungal diversity analysis, ITS I variable regions was amplified with universal primers ITS1F: CTTGGTCATTTAGAGGAAGTAA and ITS2: GCTGCGTTCTTCATCGATGC. The quality of the resulting amplicons was assessed through gel electrophoresis. Following this, the amplicons were purified using AMPure XP beads (Agencourt, USA) and subjected to a second round of PCR amplification. After a subsequent purification step with AMPure XP beads, the final amplicons were quantified using the Qubit dsDNA Assay Kit (Thermo Fisher Scientific, USA). Equal quantities of the purified amplicons were then combined for downstream sequencing. Sequencing was performed by the OE Biotech Company (Shanghai, China) on a NovaSeq 6000 System (Illumina, USA) with 250 bp paired-end reads.

### RNA-seq with unique molecular identifiers (UMIs)

To explore the differences in downstream targets and pathways of AMs between Abx and Abx + AF mice, RNA-seq was performed using unique molecular identifiers (UMIs) (Seqhealth Technology Co. Ltd., Wuhan, China). A comprehensive description of the methods can be found in the supplementary materials. Raw data have been deposited to National Center for Biotechnology Information (NCBI) under the BioProject number PRJNA1230693.

### Additional methods

Additional methods employed in this study, including immunohistochemistry; immunofluorescence staining; quantification of gut bacteria and fungi; flow cytometry analysis; multiple cytokines detection assay; western blotting; enzyme-linked immunosorbent assay (ELISA); and the isolation of AMs, are described in detail in the Supplementary Materials. Antibodies used are listed in Supplementary Materials (Table S2). The gating strategy for flow cytometry is detailed in Figure S5.

### Statistical analysis

Data are presented as mean ± SEM unless otherwise specified. Statistical analyses were performed using GraphPad Prism 10.0. For normally distributed data, comparisons between two groups were analyzed using unpaired student’s *t*-tests, whereas multiple groups were compared using one-way or two-way ANOVA followed by Tukey’s post hoc test. Non-normally distributed data were analyzed using Mann–Whitney *U* tests (two groups) or Kruskal-Wallis tests with Dunn’s correction (multiple groups). Survival curves were generated using the Kaplan–Meier method and differences were assessed by log-rank (Mantel-Cox) tests. Spearman’s rank correlation was applied to evaluate associations between fungal abundance and hospitalization duration. All statistical tests were two-tailed and a *P* value <0.05 was considered statistically significant.

## Results

### Use of antibiotics depletes gut bacteria and leads to expansion of gut fungi in patients with pneumonia

**Figure 1:**
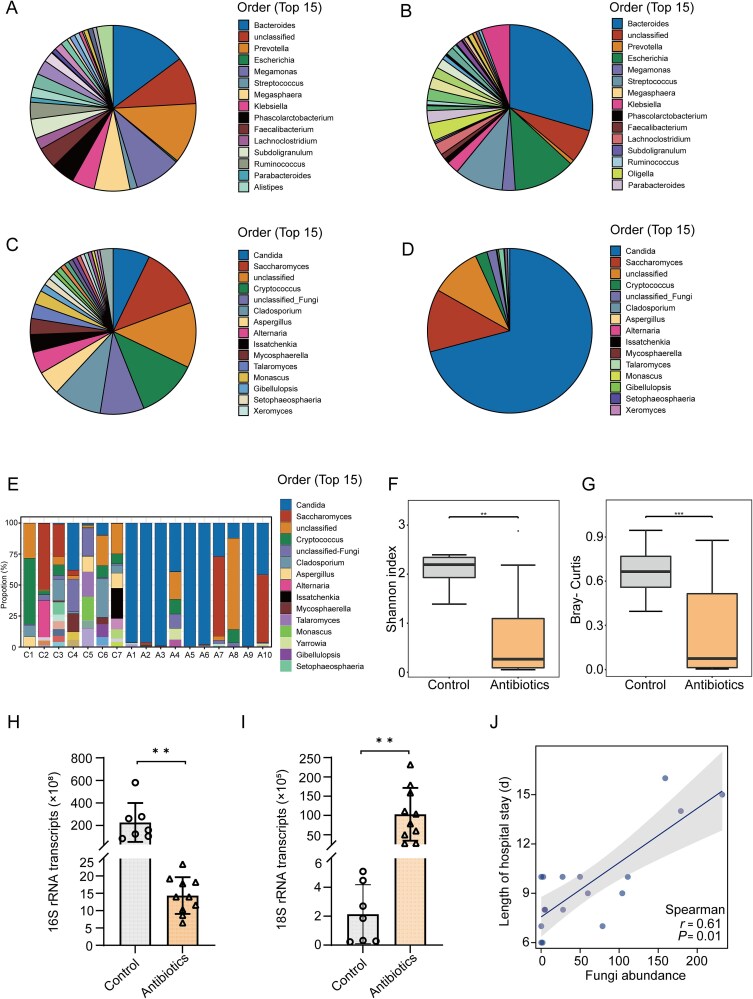
Ineffective antibiotic treatment before admission leads to overgrowth of gut fungi in CAP patients. (A, C) Overall display of gut bacteria (A) and fungi (C) at the order level in CAP patients who did not receive antibiotic treatment (n = 7). (B, D) Overall display of gut bacterial (B) and fungi (D) at the order level in CAP patients who received antibiotic treatment (n = 10). E) The composition and relative abundance of gut fungi in each CAP patient at the order level. (F, G) Comparison of alpha (F) and beta (G) diversity of gut fungi in two groups of CAP patients. (H, I) DNA from fecal samples obtained from the two groups of CAP patients was examined by quantitative PCR for bacterial 16S (H) and fungal 18S (I) rRNA genes. (J) The correlation between gut fungal abundance and the length of hospital stay in CAP patients was assessed through Spearman correlation analysis (*P* = .01, *r* = 0.61). ^**^P < .01, ^***^P < .001.

To determine the effects of antibacterial drugs on gut fungi while depleting gut bacteria, we analyzed changes in the composition of both gut bacteria and fungi. As expected, the use of antibacterial drugs significantly altered the composition of the patients’ gut bacteria (Fig. 1A and 1B). Compared with the control group (Fig. 1C), patients in the antibiotics group also showed significant changes in the composition of gut fungi (Fig. 1D). Next, we specifically examined the composition and relative abundance of gut fungi in each patient in the two groups. A decrease in gut fungal species was accompanied by an expansion of *Candida* spp. in 90% patients (9/10) of the antibiotics group (Fig. 1E). Moreover, both α (Fig. 1F) and β (Fig. 1G) diversity of gut fungi were significantly reduced in the antibiotics group compared with the control group. Quantitative PCR (qPCR) of microbial rRNA revealed that use of antimicrobial drugs depleted gut bacteria in the antibiotics group, but gut fungi expanded significantly (Fig. 1H and 1I). In addition, the hospitalization time in CAP patients was significantly positively correlated with the abundance of gut fungi (Fig. 1J). Together, these data suggest that antibacterial drugs deplete gut bacteria while promoting expansion of gut fungi, which may contribute to disease worsening in CAP patients.

### Depletion of gut fungi alleviates *K. Pneumoniae* pneumonia in Abx-treated mice

**Figure 2:**
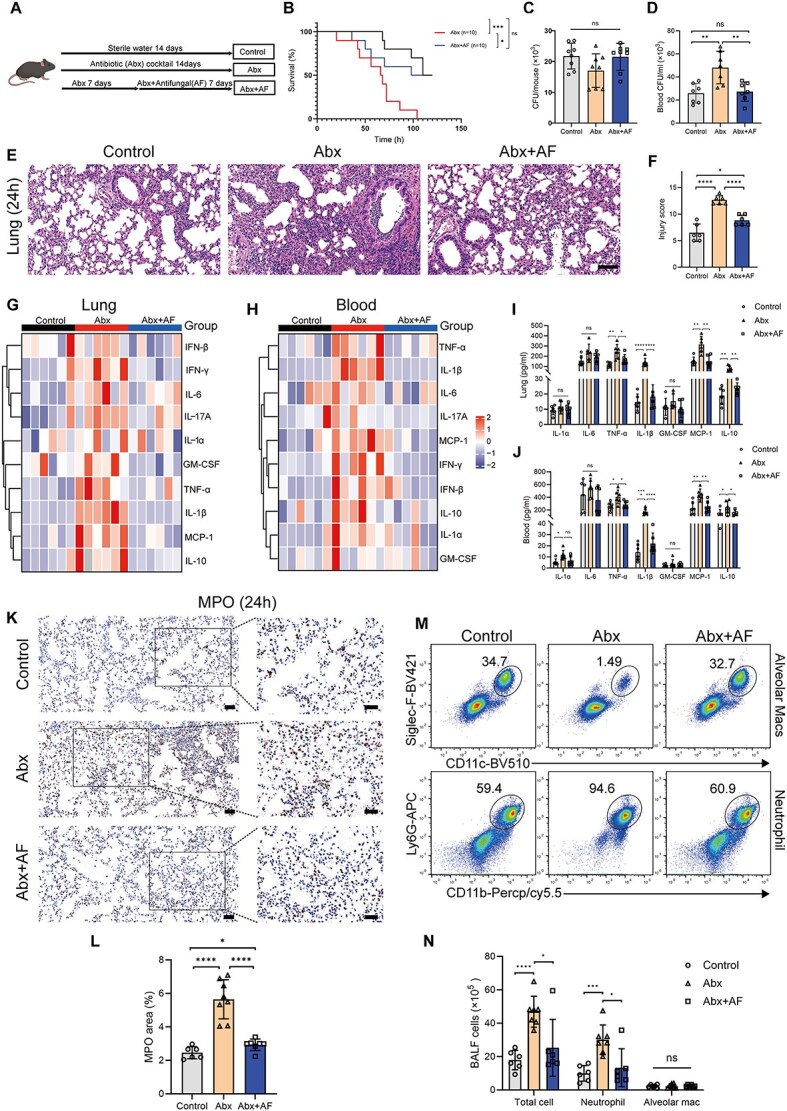
Addition of antifungal drugs (Abx + AF) alleviates *K. pneumoniae* pneumonia in Abx mice. (A) Mice were divided into the control group, Abx group, and Abx + AF group according to different treatment methods. (B-J) Abx + AF treatment significantly improved the survival rate (n = 10 in each group) (B), blood bacterial count (D), lung tissue damage score (scale bar, 100 μm) (E, F), and pro-inflammatory cytokine levels in lung tissue (G, I) and serum (H, J) of Abx-treated mice after intranasal infection with *K. pneumoniae* (K, L). Although no significant differences were observed in lung bacterial count among the three groups (C). Immunohistochemical staining of myeloperoxidase (MPO) in lung tissues of the three groups of mice infected with *K. pneumoniae* (scale bar, 50 μm for the original images, 25 μm for enlarged images)*.* (M, N) Flow cytometry analysis of BALF after 24 h of intranasal infection with *K. pneumoniae* in the three groups of mice. ^*^P < .05, ^**^P < .01, ^***^P < .001, ^***^P < .0001.

Depletion of gut fungi alleviates *K. pneumoniae* pneumonia in Abx-treated mice. Our clinical data indicated that antibacterial treatment effectively depleted gut bacteria, but it also led to expansion of gut fungi. Our previous studies have shown that Abx treatment exacerbates pneumonia symptoms in mice infected with *K. pneumoniae* intranasally [16, 17]. To verify the role of expanded gut fungi, mice in the control, Abx, and Abx + AF groups were infected intranasally with *K. pneumoniae* (Fig. 2A). We found that the addition of antifungal treatment significantly improved the severity of *K. pneumoniae* pneumonia in Abx-treated mice, including improved survival (Fig. 2B), reduced blood bacterial load (Fig. 2D), and decreased lung tissue damage (Fig. 2E and 2F). Furthermore, we excluded the therapeutic effect of antifungal treatment on *K. pneumoniae* pneumonia (Figs. S1A–S1E). Similarly, the addition of antifungal drugs reduced the levels of multiple proinflammatory cytokines in the lungs (Fig. 2G) and blood (Fig. 2H) of Abx-treated mice infected with *K. pneumoniae* via intranasal drip. Among them, IL-1β showed the most significant difference (Fig. 2I and 2J). Myeloperoxidase (MPO) is a critical pro-inflammatory enzyme in the innate immune defense against invading pathogens and is primarily secreted by activated neutrophils and monocytes [25]. When the body is infected by external pathogens, the tissue MPO content reflects the intensity of the inflammatory response to some extent. We performed MPO immunohistochemical staining on lung tissues from the three groups of mice 24 h after intranasal infection with *K. pneumoniae*. MPO content in the lung tissue of mice in the Abx + AF group was significantly lower than that in the Abx group 24 h after infection with *K. pneumoniae* (Fig. 2K and 2L). To better understand how expanded gut fungi influence the lung’s immune response to external pathogens, we collected bronchoalveolar lavage fluid (BALF) from the three groups of mice 24 h after infection for flow cytometry analysis. 24 h after intranasal infection with *K. pneumoniae*, the number of neutrophils in the BALF of Abx mice was significantly higher than that in the control group and the addition of antifungal drugs (Abx + AF) significantly reduced the number of neutrophils in the BALF of Abx mice. In addition, there was no significant difference in the number of AMs in the BALF of the three groups of mice after infection (Fig. 2M and 2N). These results suggest that the addition of antifungal drugs can alleviate the inflammatory response of Abx mice after intranasal infection with *K. pneumoniae.*

### Depletion of gut bacteria in mice leads to the expansion of gut fungi

**Figure 3:**
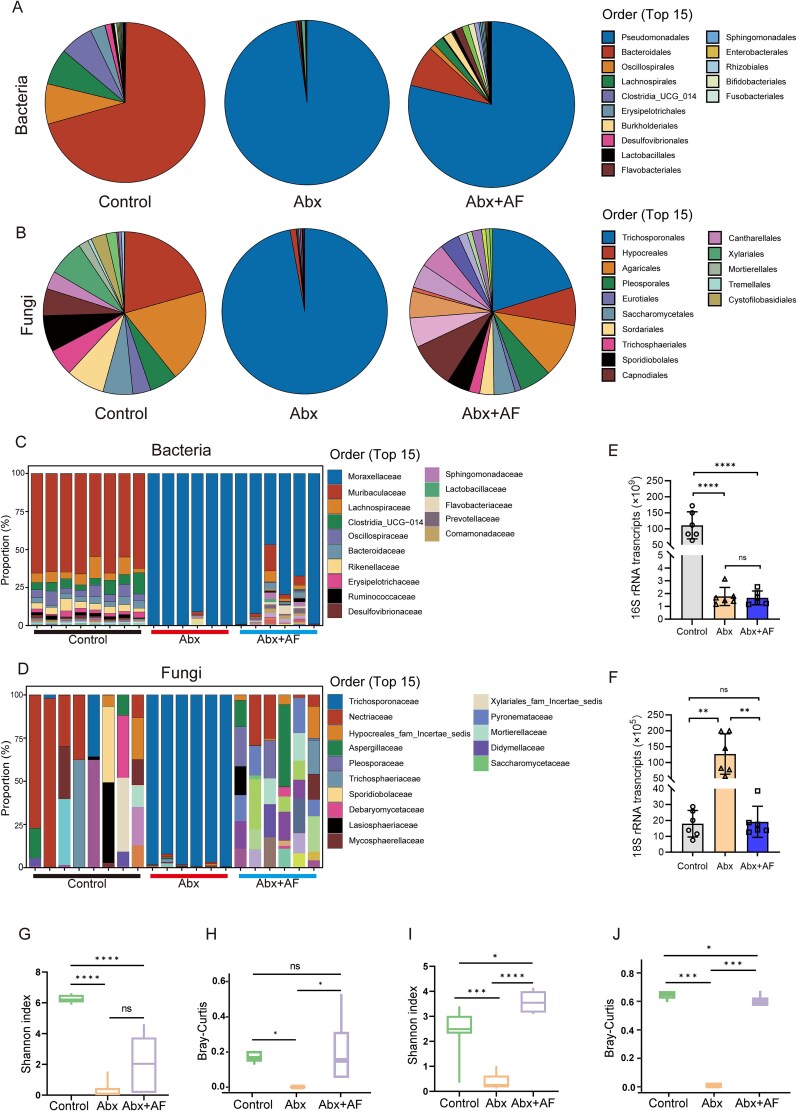
16S rRNA genes and ITS genes sequencing analysis of gut microbiota in control, Abx, and Abx + AF group mice. (A, B) The pie charts display the composition of gut bacteria (A) and fungi (B) in the three groups of mice. C, D) the composition and relative abundance of gut bacteria (C) and fungi (D) in each mouse from the three groups. (E, F) DNA from fecal samples obtained from the three groups of mice was examined by qPCR for bacterial 16S (E) and fungal 18S (F) rRNA genes. (G-J) Comparison of alpha (G) and beta (H) diversity of gut bacteria, and alpha (I) and beta (J) diversity of gut fungi in the three groups of mice. ^*^P < .05, ^**^P < .01, ^***^P < .001, ^****^P < .0001.

To further investigate the impact of antibiotics on gut fungi, we conducted 16S rRNA and ITS sequencing on fecal samples from control, Abx, and Abx + AF groups. Antibiotic treatment reshaped the composition of both gut bacteria and fungi (Fig. 3A, 3B). Although Abx treatment reduced gut fungal diversity, subsequent AF agent administration partially restored fungal diversity in these mice. We characterized the composition and relative abundance of gut bacteria (Fig. 3C) and fungi (Fig. 3D) in individual mice from the control, Abx, and Abx + AF groups. Comparative analysis of α-diversity and β-diversity indices revealed that antibiotic treatment significantly decreased both α- and β-diversity of gut bacteria, whereas subsequent antifungal administration partially reversed this reduction (Fig. 3E–H). Similarly, Abx treatment significantly reduced both α-diversity (Fig. 3I) and β-diversity (Fig. 3J) of gut fungi. The addition of antifungal drugs substantially increased fungal α- and β-diversity compared to Abx-treated mice. We further performed qPCR analysis of gut bacterial and fungal loads across all three groups. Mirroring clinical observations, Abx treatment induced a dramatic fungal expansion concomitant with bacterial depletion in the murine gut. Antifungal administration effectively reversed these dysbiotic changes, restoring fungal populations to baseline levels (Fig. 3E, 3F). In summary, although Abx treatment significantly reduced both the abundance and diversity (including α- and β-diversity) of gut bacteria, it simultaneously increased fungal abundance while decreasing fungal diversity (in both α- and β-diversity) in mice. The addition of antifungal treatment (Abx + AF) effectively suppressed the Abx-induced fungal overgrowth and partially restored gut fungal diversity (α- and β-diversity) compared to Abx-treated mice.

### Abx treatment upregulates the expression of Dectin-1 in mouse alveolar macrophages

**Figure 4:**
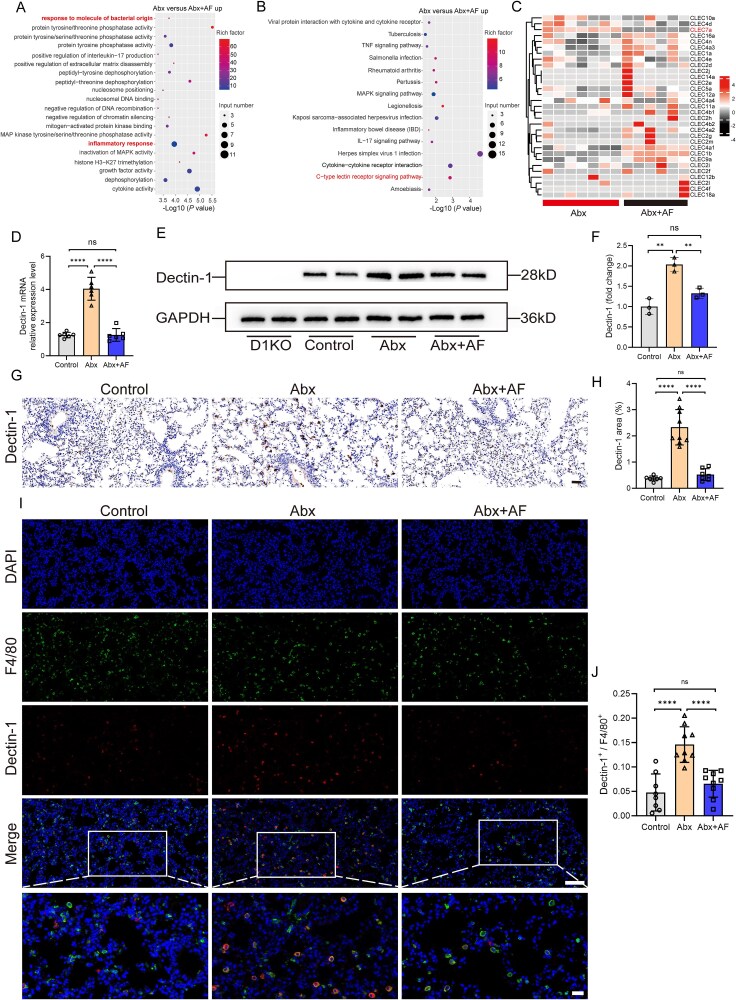
Abx treatment upregulates Dectin-1 expression in mouse AMs. (A-C) RNA-seq was performed on AMs of Abx and Abx + AF mice stimulated with LPS for 6 h *in vitro*. GO enrichment (A) and KEGG enrichment (B) of cluster-specific marker gene transcripts upregulated in Abx versus Abx + AF AMs. (C) Heatmap displaying all C-type lectin receptor family gene transcripts in AMs of Abx and Abx + AF mice. D) Dectin-1 expression in lung tissue of control, Abx, and Abx + AF mice was analyzed by qPCR. E, F) Dectin-1 protein expression in lung tissue of D1KO, control, Abx, and Abx + AF mice. G, H) representative immunohistochemical analyses of Dectin-1 in lung tissue of control, Abx, and Abx + AF mice (scale bar, 50 μm). I, J) representative dual-immunofluorescence staining of Dectin-1 and F4/80 in lung tissue of control, Abx, and Abx + AF mice (scale bar, 100 μm for the original images, 25 μm for enlarged images). ^**^P < .01, ^****^P < .0001.

AMs are the first line of defense of the respiratory system against external pathogens. When stimulated by pathogens, AMs induce inflammation by producing chemokines and cytokines (such as type I IFN, TNF-α, and IL-1β), and recruit and activate neutrophils, monocytes, and dendritic cells (DCs) [26, 27]. To better understand the effects of Abx and Abx + AF treatment on AMs in mice, we collected AMs from the Abx and Abx + AF groups for *in vitro* culture and performed RNA sequencing (RNA-seq) after LPS stimulation for 6 h. Gene ontology (GO) enrichment analysis showed that the expression of inflammation-related pathways in AMs from mice in the Abx group was upregulated compared to the Abx + AF group (Fig. 4A). Kyoto Encyclopedia of Genes and Genomes (KEGG) enrichment analysis revealed that multiple signaling pathways in the Abx group were upregulated compared to the Abx + AF group, among which the upregulation of the C-type lectin receptor (CLR) signaling pathway of particular interest because of its established role in fungal recognition and immune regulation, which aligns with our observed mycobiome alterations (Fig. 4B). Through transcriptomic sequencing of AMs, we performed comparative analysis of all expressed C-type lectin receptor genes, the expression of *Clec7a* in AMs of the Abx group was significantly higher than that in the Abx + AF group (Fig. 4C and Fig. S2). Given that *Clec7a* encodes the Dectin-1 receptor, which is a receptor for β-glucans and plays important roles in host defense against fungi and immune homeostasis of the gut [28], we examined Dectin-1 expression levels in lung tissues from the three groups of mice. We found that Abx treatment significantly upregulated the expression of Dectin-1 in the lungs of mice, whereas the addition of antifungal drugs significantly decreased the expression of Dectin-1 after Abx treatment (Fig. 4D, 4E, and  4F). Immunohistochemical analyses confirmed that Abx treatment upregulated the expression of Dectin-1 in mouse lung tissue, whereas the addition of antifungal drugs significantly reduced the high expression of Dectin-1 in the lungs of Abx-treated mice (Fig. 4G and 4H). Double-immunofluorescence staining of Dectin-1 together with F4/80 staining confirmed that Abx treatment upregulated the expression of Dectin-1 in lung F4/80+ macrophages. Similarly, the addition of antifungal drugs significantly reduced the high expression of Dectin-1 in lung F4/80+ macrophages of Abx-treated mice (Fig. 4I and 4J). Representative flow cytometry histograms of surface molecules on AMs, neutrophils, monocytes, and lymphocytes confirmed that Dectin-1 was only upregulated in the AMs of Abx mice (Fig. S3A and S3B). These data suggest that Abx treatment upregulates the expression of Dectin-1 in AMs of mice and the addition of antifungal drugs can reduce the high expression level of Dectin-1 after Abx treatment.

### Dectin-1 deficiency ameliorates lung injury after *K. Pneumoniae* infection in Abx-treated mice.

**Figure 5:**
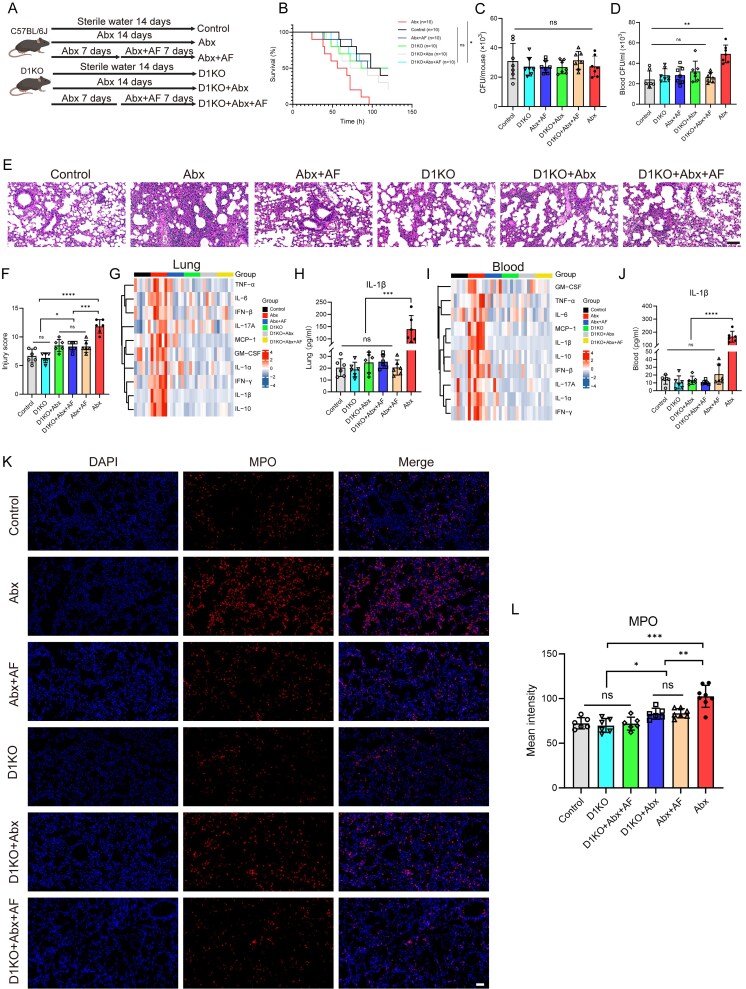
Dectin-1 deficiency ameliorated *K. pneumoniae* pneumonia in Abx mice. A) According to different treatment methods, the mice were divided into control group, Abx group, Abx + AF group, D1KO group, D1KO + Abx group and D1KO + Abx + AF group. B-L) Dectin-1 deficiency significantly improved the survival rate (n = 10 in each group) (B), blood bacterial count (D), lung tissue damage score (scale bar, 100 μm) (E, F), lung tissue (G, I) and serum (H, J) pro-inflammatory cytokine levels and lung MPO mean intensity (scale bar for all figures, 50 μm) (K, L) in Abx mice after intranasal infection with *K. pneumoniae*. ^*^P < .05, ^**^P < .01, ^***^P < .001, ^****^P < .0001.

Based on our results, we hypothesized that Abx treatment led to a large expansion of gut fungi, which upregulated the expression of Dectin-1 in mouse AMs. This, in turn, causes AMs to initiate a more severe inflammatory response after recognizing the invading *K. pneumoniae*. To elucidate the impact of Dectin-1, we used D1KO and WT C57BL/6 mice for treatment and grouping according to Fig. 5A. We found that the loss of Dectin-1 significantly improved the severity of *K. pneumoniae* pneumonia in Abx-treated mice, including improved survival (Fig. 5B), reduced blood bacterial load (Fig. 5D), and decreased lung tissue damage (Fig. 5E and 5F). Consistent with our earlier findings regarding pulmonary bacterial clearance (Fig. 2C), Dectin-1 deficiency did not significantly affect lung bacterial loads in antibiotic-treated mice (Fig. 5C). Multifactor detection was performed on lung tissue and serum from the six groups of mice infected with *K. pneumoniae* after 24 h. The loss of Dectin-1 reduced the levels of multiple proinflammatory cytokines in the lung tissue and serum of Abx-treated mice after nasal drip infection with *K. pneumoniae*, especially IL-1β (Figs. 5G–J). MPO immunofluorescence analysis showed that the loss of Dectin-1 significantly reduced the mean fluorescence intensity of MPO in lung tissue of Abx-treated mice with nasal infection of *K. pneumoniae* (Fig. 5K and 5L). Dectin-1 deficiency and antifungal drug treatment (Abx + AF) achieved the same effect on survival rate, blood bacterial load, lung tissue injury score, inflammatory factor levels, and mean fluorescence intensity of MPO in Abx-treated mice after intranasal infection with *K. pneumoniae*. Furthermore, comparative analysis revealed no significant improvements in survival rate, lung injury, neutrophil recruitment, or inflammatory cytokine levels (both in lung tissue and serum) in the D1KO + Abx + AF group relative to the D1KO + Abx group. These results provide compelling evidence that gut fungal dysbiosis exacerbates *K. pneumoniae* pneumonia specifically through the Dectin-1-mediated pathway.

### **Alveolar macrophages** in Abx-treated mice release more IL-1β and recruit more neutrophils

**Figure 6:**
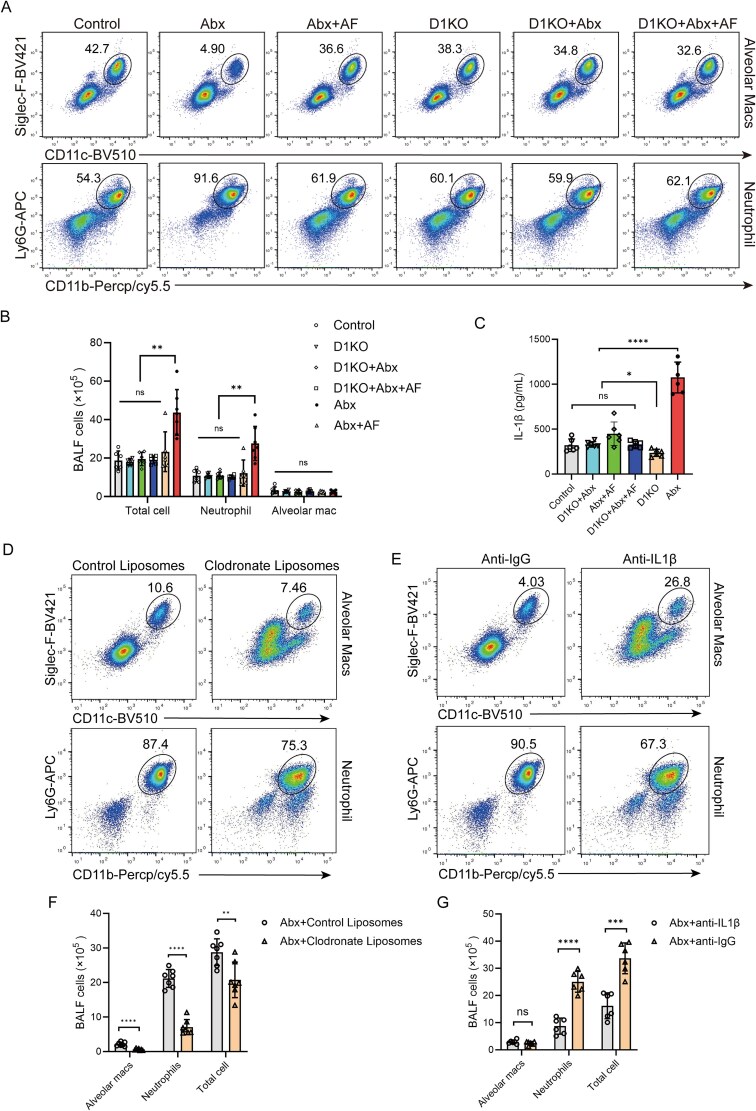
AMs in Abx mice secrete more IL-1 β to recruit more neutrophils. (A, B) Flow cytometry analysis of BALF of mice after 24 h of intranasal infection with *K. pneumoniae* in control, Abx, Abx + AF, D1KO, D1KO + Abx and D1KO + Abx + AF groups. C) IL-1β in the cell supernatant of AMs from mice in control, Abx, Abx + AF, D1KO, DIKO+Abx, D1KO + Abx + AF groups stimulated with LPS *in vitro* were detected by ELISA. (D, F) Flow cytometry analysis of BALF of mice with/without AMs depletion after infected with *K. pneumoniae.* (E, G) Flow cytometry analysis of BALF of mice with/without IL-1β neutralization after infected with *K. pneumoniae.*  ^**^P < .01, ^****^P < .0001.

The number of neutrophils in BALF was significantly increased in Abx-treated mice following intranasal *K. pneumoniae* infection compared to control, whereas Abx + AF treatment significantly reduced neutrophil counts relative to Abx-treated mice (Fig. 2). Based on these results, we hypothesized that the upregulation of Dectin-1 expression in mouse AMs after Abx treatment caused AMs to release more IL-1β after recognizing *K. pneumoniae*, thus recruiting more neutrophils. Flow cytometry analysis revealed that Dectin-1 deletion significantly reduced the number of neutrophils in the BALF of Abx-treated mice after infection with *K. pneumoniae*, and the effect was similar to that of Abx + AF treatment (Fig. 6A and 6B). This result confirms the role of Dectin-1. To verify the role of AMs, we treated Abx mice with clodronate liposomes and control liposomes. We successfully depleted AMs in Abx mice (Fig. S4). We then found that the number of neutrophils in the BALF of Abx mice in the AM-depleted group was significantly lower than that in the non AM-depleted group after intranasal infection with *K. pneumoniae* (Fig. 6D and 6F). This result confirms the role of AMs.

We explored the role of IL-1β. We collected AMs from mice in the control, Abx, Abx + AF, D1KO, D1KO + Abx and D1KO + Abx + AF groups for *in vitro* culture. After stimulation with LPS (100 ng/ ml) for 6 h, we collected the cell supernatant and detected IL-1β by ELISA. We found that AMs from mice in the Abx group secreted more IL-1β after LPS stimulation. Compared to the Abx + AF group, the D1KO + Abx and D1KO + Abx + AF groups showed no significant differences in LPS-stimulated IL-1β production by AMs, confirming that Dectin-1 knockout suppresses AM hyperactivation (Fig. 6C). To validate our hypothesis, antibiotic-treated mice were administered either IL-1β neutralizing antibody or control IgG antibody prior to intranasal *K. pneumoniae* challenge. Flow cytometric analysis demonstrated that IL-1β neutralization significantly attenuated neutrophil infiltration in BALF compared to IgG-treated controls (Fig. 6E and 6G).

## Discussion

The rising prevalence of antibiotic resistance in *K. pneumoniae* is primarily driven by the widespread misuse of antimicrobial agents. In clinical practice, managing infections caused by drug-resistant *K. pneumoniae* strains typically requires the use of higher-tier antibiotics or escalated therapeutic regimens. Ineffective antibiotic therapy not only prolongs disease duration but also induces significant gut microbiota dysbiosis in patients. Through continuous crosstalk with host immunity, the gut microbiota precisely calibrates inflammatory responses by controlling both the amplitude and phenotypic characteristics of immune reactions [29]. Emerging evidence indicates that the gut-lung axis*—*the reciprocal interaction between gut microbiota and respiratory system—plays a pivotal role in shaping lung immunity via multiple pathways [30]. Although accumulating evidence has established associations between gut microbial dysbiosis and both immune dysregulation and pulmonary pathogenesis, current research on the gut-lung axis remains predominantly bacterio-centric, overlooking potential contributions from other gut microbiota components.

Gut fungi, though numerically minor constituents of the intestinal microbiota, are increasingly recognized as critical regulators of host homeostasis and disease pathogenesis [31]. These eukaryotic microorganisms persist in a bacteriocentric gut ecosystem, where bacterial communities exert profound influence over fungal colonization dynamics, metabolic activity, and host–microbe interactions [32]. Normal gut bacteria provide colonization resistance against fungi, preventing excessive colonization of gut fungi. Previous studies have confirmed that the use of broad-spectrum antibiotics is associated with the expansion of gut fungi in patients, increasing the risk of invasive fungal infections that migrate from the intestine [33]. The same phenomenon has also been observed in mice [34]. Both our clinical data and animal experiments confirm that antibiotics disrupt the homeostasis of gut bacteria and lead to the expansion of gut fungi. Dysbiosis of gut fungi has been linked to various intestinal diseases, such as inflammatory bowel disease [35, 36], irritable bowel syndrome [37], coeliac disease [38], and colorectal cancer [39, 40]. In addition to intestinal cancer, dysbiosis of gut fungi has been shown to be associated with various extraintestinal cancers, such as pancreatic ductal adenocarcinoma [41], hepatocellular carcinoma [42] and nonsmall cell lung cancer [43]. Growing experimental and clinical evidence demonstrates that gut fungi can exert systemic modulation of pulmonary antifungal immunity via the gut-lung axis [44]. This finding demonstrates that gut fungi modulate the lung’s immune defense against respiratory pathogens. In addition, we identified a significant positive correlation between gut fungal abundance and hospitalization duration in CAP patients, indicating that fungal overgrowth may contribute to disease severity. Therefore, we hypothesized that the expansion of gut fungi after Abx treatment may also contribute to the exacerbation of *K. pneumoniae* pneumonia in mice.

The fresh air we breathe every day may contain pathogens and dust [45]. AMs, as the first line of defense in the respiratory system, continuously capture and engulf these pathogens without causing excessive inflammatory reactions [46]. When the phagocytic function of AMs is surpassed, AMs initiate inflammation through the production of chemokines and cytokines (such as IL-1β and type I IFNs), which recruit and activate neutrophils, monocytes, and DCs [47]. Our results showed that fluconazole supplementation in Abx-treated mice both corrected gut fungal dysbiosis and ameliorated *K. pneumoniae* pneumonia. Abx treatment elevated inflammatory factors in murine lung and serum, which were significantly reduced by antifungal treatment, with IL-1β demonstrating the most pronounced reduction. BALF analysis revealed higher neutrophil counts in antibiotic-treated mice compared to control and Abx + AF groups, consistent with their more severe pneumonia symptoms and enhanced lung inflammation. At 24 h post intranasal infection, Abx-treated mice showed reduced pulmonary bacterial CFU (not statistically significant) but significantly increased blood CFU relative to controls (*P* < .01). This pattern resulted from neutrophil-mediated damage to alveolar-epithelial and pulmonary-endothelial integrity, promoting bacterial translocation into circulation [48]. Abx-treated mice exhibited elevated proinflammatory cytokines (especially IL-1β) in lung and serum after infection, while Dectin-1 knockout mice produced significantly less IL-1β under identical conditions. These findings support our hypothesis and confirm previous clinical observations.

In summary, we found that antibacterial drugs deplete gut bacteria in mice and also cause gut fungi to expand. The expanded gut fungi further upregulate the expression of Dectin-1 in mouse AMs, which, in turn, causes AMs to release more IL-1β after recognizing *K. pneumoniae*, thereby recruiting more neutrophils and leading to a stronger inflammatory response. Our study highlights the role of gut fungi in *K. pneumoniae* pneumonia, providing new insights for the clinical treatment of drug-resistant *K. pneumoniae* pneumonia. In addition, we must acknowledge that our study has several further limitations. First, this was a single-center study with a relatively small clinical cohort size. Second, we did not account for potential influences from the pulmonary microbiome. Although these microbial communities represent a minor proportion, they may still exert certain biological functions. These aspects represent important directions for future investigation.

## Supplementary Material

supplementary_materials_2_wraf181

## Data Availability

Raw data of 16S rRNA genes and ITS sequences have been deposited to National Center for Biotechnology Information (NCBI) under the BioProject number PRJNA1230691 and PRJNA1230689. Raw data of RNA-seq with UMIs have been deposited to the National Center for Biotechnology Information (NCBI) under BioProject number PRJNA1230693. The datasets generated and/or analyzed during the current study are available at https://doi.org/10.5281/zenodo.16813777.
