# Supplementary material for: Dysbiotic gut fungi exacerbate Klebsiella pneumoniae lung infection via Dectin-1-mediated alveolar macrophage hyperactivation
Source: ISME J. 2025 Aug 16;19(1):wraf181. doi: 10.1093/ismejo/wraf181 (PMC12422099; doi:10.1093/ismejo/wraf181)
Supplement: supplementary_materials_2_wraf181 [file supplementary_materials_2_wraf181.docx]

| **Characteristics** | **Antibiotics (n=10)** | **Control (n=7)** | ***P* value** |
| --- | --- | --- | --- |
| Age (years) | 68.40 ± 10.98 | 70.86 ± 6.38 | 0.6243 |
| Sex |  |  |  |
| Male | 7 (70.00%) | 4 (57.10%) | 0.9758 |
| Female | 3 (30.00%) | 3 (42.90%) |  |
| BMI (kg/m^2^) | 24.66 ± 1.28 | 23.39 ± 1.19 | 0.0691 |
| Smoking status |  |  |  |
| Never smoker | 5 (50.00%) | 5 (71.43%) | 0.1910 |
| Former smoker | 0 (0%) | 1 (14.29%) |  |
| Current smoker | 5 (50.00%) | 1 (14.29%) |  |
| WBC (×10^9^) | 10.45 ± 2.55 | 8.16 ± 1.44 | 0.0617 |
| \|N\| (×10^9^) | 7.93 ± 2.01 | 5.89 ± 1.32 | 0.0433 |
| CRP (mg/L) | 74.13 ± 32.80 | 48.03 ± 24.91 | 0.1159 |
| Length of hospital stay (d) | 10.80 ± 2.93 | 7.86 ± 1.55 | 0.0373 |

**Supplementary Table 1**. Demographic and clinical features of patients who provided fecal specimens

**Supplementary Table 2**. Antibodies used in experiments

| Antibody | Company | Catalog number |
| --- | --- | --- |
| Dectin-1 (WB) | LifeSpan Bioscience | LS-B8826 |
| Dectin-1 (IHC) | Abcam | ab300497 |
| MPO | Abcam | ab208670 |
| F4/80 | Abcam | ab300421 |
| GAPDH | Cell Signaling Technology | 5174 |
| Goat Anti-Rabbit IgG (HRP) | Abcam | Ab6721 |

**Generation of Clec7a Knockout Mouse Model**

*Clec7a* knockout mouse model used in this study was generated by Shanghai Model Organisms Center, Inc (Shanghai, China). CRISPR/Cas9 system was applied to achieve the deletion of specific gene segments, resulting in a functional loss of *Clec7a*. Detailed procedure was as follows: Four gRNAs targeting exon 2-3 of *Clec7a* were designed based on the gene sequence. The gRNAs sequences are:

• gRNA1: 5’-CTGTGACCAAGTAGGACTTCAGG-3’

• gRNA2: 5’-TGTGACCAAGTAGGACTTCAGGG-3’

• gRNA3: 5’-TACTGAGGAAGGCAAAGAGCTGG-3’

• gRNA4: 5’-TGGATCTGTCTGAGCTAAGATGG-3’

In vitro-transcribed Cas9 mRNA and gRNAs were microinjected into zygotes of C57BL/6J mice. The zygotes were then transferred to pseudopregnant female mice to obtain F0-generation offspring. Positive F0 mice were crossed with wild-type C57BL/6J mice to produce F1 heterozygous *Clec7a* knockout mice. The genotypes of F1 mice were verified using PCR and sequencing. Male and female F1 heterozygous mice were intercrossed to generate homozygous *Clec7a* knockout mice.

**Quantification of gut bacteria and fungi**

Initially, genomic DNA was extracted from fecal samples using the QIamp DNA Stool Mini Kit, followed by evaluation of the extraction efficiency using 1.2% agarose gel electrophoresis. Subsequently, for the preparation of qPCR templates, plasmid DNA was extracted using the Axygen plasmid miniprep Kit (Axygen), and its concentration was determined. Thirdly, real-time quantitative PCR (qPCR) was performed with the primers (Bacteria: F: ACTCCTACGGGAGGCAGCAGT and R: ATTACCGCGGCTGCTGG; Fungi: F: GGRAAACTCACCAGGTCCAG and R: GSWCTATCCCCAKCACGA). The PCR reaction mixture included SYBR Premix Ex Taq™ (2x), forward primer F (10 μM), reverse primer R (10 μM), DNA template, and ddH₂O, to a total volume of 25 μl. The PCR amplification protocol was as follows: 95°C for 30 s for initial denaturation, followed by 40 cycles of 95°C for 10 s, 60°C for 30 s, and 72°C for 30 s. The experimental data were collected using the standard laboratory real-time PCR machine, FTC-3000.

**Histopathological evaluation of lung injury**

Three random tissue sections from four different lungs in each group were examined by a pathologist who was blinded to the genetic background/treatment of the mice. Lung injury was scored according to the following criteria: 1) alveolar congestion, 2) hemorrhage, 3) infiltration or aggregation of neutrophils in airspace or vessel wall and 4) thickness of the alveolar wall/hyaline membrane formation. For each subject, a 5-point scale was applied: 0, minimal (little) damage; 1+, mild damage; 2+, moderate damage; 3+, severe damage; and 4+, maximal damage. Points were added up and are expressed as median ± range of injury score [1].

**RNA-seq with unique molecular identifiers (UMIs)**

Briefly, 2 µg of total RNA extracted from alveolar macrophages of Abx and Abx+AF mice was used for stranded RNA-seq library preparation (Catalogue #DR08502; KC-Digital™ Stranded mRNA Library Prep Kit for Illumina, Seqhealth Technology Co. Ltd.), following the manufacturer’s instructions. The kit eliminates duplication bias in PCR and sequencing steps by using the UMIs of eight random bases to label the pre-amplified cDNA molecules. Library products corresponding to 200–500 bps were enriched, quantified, and sequenced on a NovaSeq 6000 sequencer (PE150 model) (Illumina, San Diego, CA, USA). Raw sequencing data were filtered using Trimmomatic (version 0.36). Clean reads were further treated with in-house scripts to eliminate duplication bias introduced in library preparation and sequencing. TopHat2 (version 2.0.13) was used to align de-duplicated reads to the reference genome (GRCh38/hg38). The R package “DESeq2” (version 2.15.13) was used to identify differentially expressed genes between groups. Genes with |log2(fold-change)| >1 and FDR<0.05 were considered to be differentially expressed. The molecular functions and pathways of dysregulated genes were analysed in KOBAS 2.0. Raw data have been deposited to National Center for Biotechnology Information (NCBI) under the BioProject number PRJNA1230693.

**Immunohistochemistry**

Paraffin-embedded mouse lung tissue slides underwent heat-induced antigen retrieval and peroxidase blocking to eliminate endogenous hydrogen peroxide. After blocking with 3% bovine serum albumin(BSA) in PBS, the slides were incubated with primary antibodies in a humidified chamber at 4°C overnight. They were then incubated with horseradish peroxidase-labeled secondary antibodies at room temperature for 30 minutes and stained with 3,3’-diaminobenzidine substrate solution.Antibodies used are listed in Table S2.

**Immunofluorescence staining**

Paraffin-embedded mouse lung tissue slides underwent heat-induced antigen retrieval and the endogenous peroxidase activity was blocked by incubating the sections with 3% H_2_O_2_ at room temperature. The sections were then blocked with 3% BSA, primary antibody incubation was performed overnight at 4°C. Afterward, sections were incubated with HRP-conjugated secondary antibody for 50 minutes at room temperature. Signal amplification was achieved using 1× dye working solution (with TSA fluorescence dye) for 10 minutes, followed by PBS washing. After a final microwave treatment, sections were washed and mounted with DAPI for nuclear staining, followed by observation under a fluorescence microscope. Antibodies used are listed in Table S2.

**Western bloting**

Total protein was extracted from mouse lung tissue using RIPA lysis buffer containing 1% PMSF and phosphatase inhibitors, and transferred onto a polyvinylidene fluoride (PVDF) membrane. The membrane was blocked at room temperature for 1.5 hours with 5% non-fat dry milk diluted in TBS-T. After overnight incubation at 4°C with the appropriate primary antibody, the membrane was washed three times with TBS-T. The membrane was then incubated with the secondary antibody at room temperature for 1 hour, followed by three washes with TBS-T. Protein detection was performed using the ECL Plus Western blotting detection system. Additionally, the content of glyceraldehyde-3-phosphate dehydrogenase (GAPDH) was used as an internal control. Antibodies used are listed in Table S2.

**Quantitative real-time PCR**

Total RNA from the lung tissues was isolated using the TRIzol reagent (Takara, Japan) in accordance with the provided protocol. cDNA was synthesized using a cDNA synthesis kit (Takara, Japan) according to the manufacturer’s instructions. For mRNA detection, GAPDH served as an internal control. Real-time PCR was conducted using SYBR Green (TaKaRa, Japan) with a three-step real-time PCR system (Light Cycler 96, Roche). Primer sequences are provided as below:

| Gene | Forward primer (5’ to 3’) | Reverse primer (5’ to 3’) |
| --- | --- | --- |
| GAPDH | CATCACTGCCACCCAGAAGACTG | ATGCCAGTGAGCTTCCCGTTCAG |
| Dectin-1 | GGGTGCCCTAGGAGGTTTTT | TGCTGATCCATCCTCCCAGA |

**Isolation of alveolar macrophages**

Mice were sacrificed and immediately exsanguinated. Bronchoalveolar lavage fluid (BALF) was collected with 4 ml of 37°C sterile PBS containing 1 mM EDTA. Cells were pelleted and resuspended in RPMI 1640 supplemented with 5.0% (vol/vol) FBS and then were allowed to adhere to a tissue culture flask for 2 h (37°C, 5% CO2 [vol/vol]). In general, alveolar macrophage purity was more than 93% as analyzed by flow cytometry (FACS Celesta; BD Biosciences).

**Alveolar macrophages depletion in mouse model**

AMs were depleted by intranasal administration of clodronate liposomes (50μl per mouse; YEASEN, Shanghai, China). The control group was treated by control liposomes (50 μl per mouse; YEASEN, Shanghai, China).

**In vivo IL-1β neutralization**

The IL-1β neutralization method for mice was carried out as previously described[2]. In the experimental group, mice were injected via the tail vein with 20 μg of IL-1β neutralizing antibody (R&D Systems, AF-401-NA) 3 hours prior to infection. The control group mice were injected with an equivalent amount of normal goat IgG control antibody (R&D Systems, AB-108-C).

**Flow cytometry**

For flow cytometry, cells from BALF were collected as previously described and resuspended in Cell Staining Buffer (420201) to create a single-cell suspension. Fc receptors were blocked by incubating the cells with TruStain FcX™ (Clone: 93, 101320) at 4°C for 15 minutes. The following surface fluorochrome-conjugated antibodies were then used: CD45 BV785 (Clone: 30-F11, 103149), CD11b PerCP-Cy5.5 (Clone: M1/70, 101228), CD11c BV510 (Clone: N418, 117338), Siglec-F BV421 (Clone: S17007L, 155509), Ly-6G APC (Clone: 1A8, 127614), Ly-6C FITC (Clone: HK1.4, 128005), and Dectin-1 PE (Clone: RH1, 144304). The cells were stained at 4°C for 45 minutes. Zombie NIR dye (423105) was then added for 15 minutes to distinguish live and dead cells. Afterward, cells were centrifuged at 350g for 5 minutes, resuspended in Cell Staining Buffer to form a single-cell suspension, and prepared for analyzed by a FACSCalibur flow cytometer (FACS Celesta, BD Biosciences). All reagents were purchased from BioLegend.

**ELISA and multiple cytokines detection assay**

Mouse IL-1β ELISA kit (1210122, Dakewe Bio-engineering Co. Ltd, Shenzhen, China) was used to measure the interleukin-1 beta (IL-1β) concentrations in cell supernatant. Interleukin-1 alpha (IL-1α), IL-1β, interleukin-6 (IL-6), interleukin-10 (IL-10), interferon beta (IFN-β), interleukin-17A (IL-17A), monocyte chemoattractant protein 1 (MCP-1), tumor necrosis factor alpha (TNF-α), interferon-gamma (IFN-γ), and granulocyte macrophage colony stimulating factor (GM-CSF) were measured from lung sonicates and serum using LEGENDplex Mouse Inflammation Panel (BioLegend, 740150) according to manufacturer instructions.


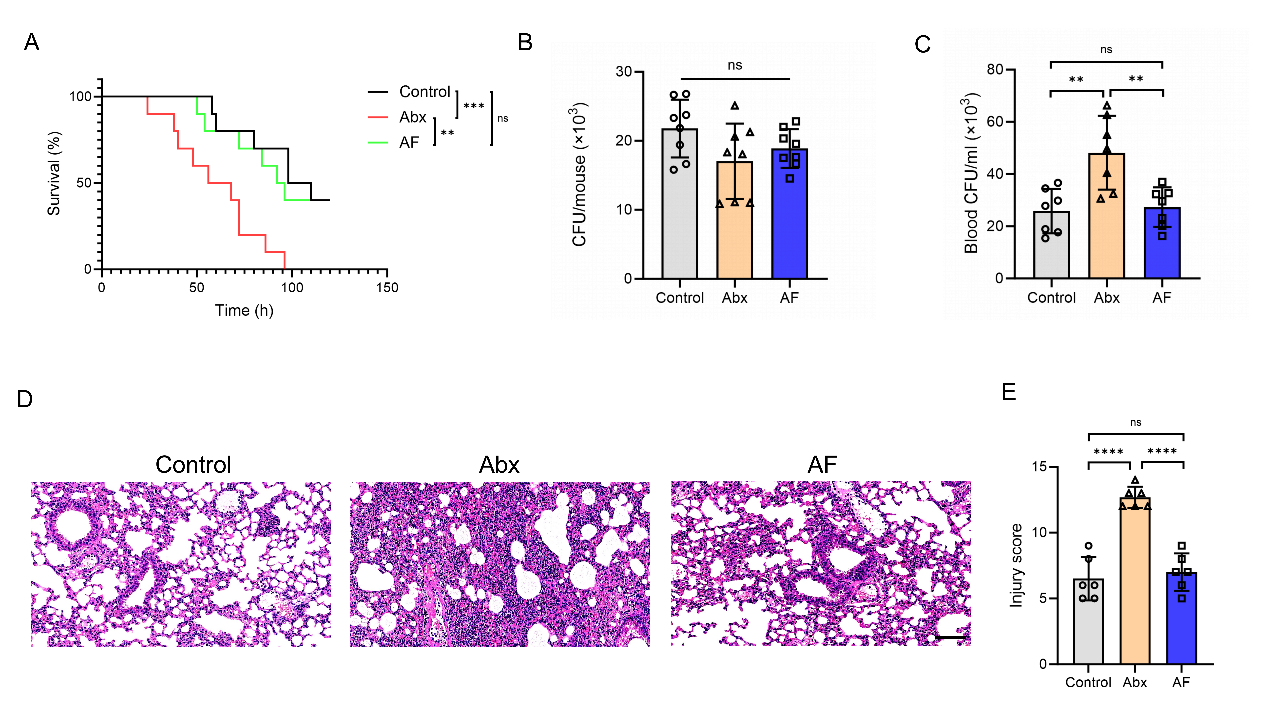


Figure S1. Antifungal treatment showed no therapeutic effect on *K.pneumoniae* pneumonia in mice. Compared to the control group, antifungal treatment did not improve the survival rate(A), lung bacterial count (B), blood bacterial count (C), lung tissue damage score (D, E, scale bar, 50μm). ** *P*< 0.01, *** *P*< 0.001, **** *P*< 0.0001.


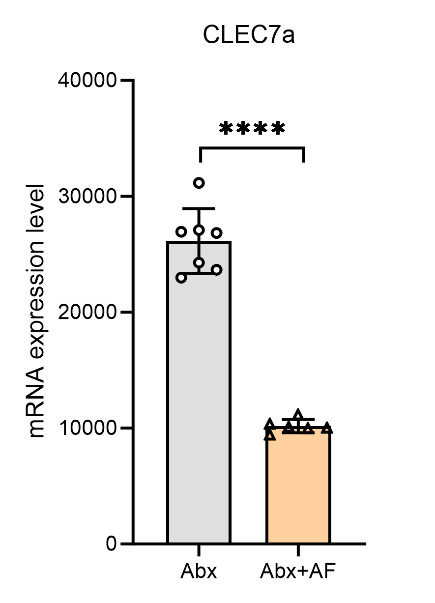


Figure S2. RNA-Seq revealed that Clec7a mRNA expression level of AMs in Abx mice was significantly higher than that in Abx+AF mice. **** *P*< 0.0001.


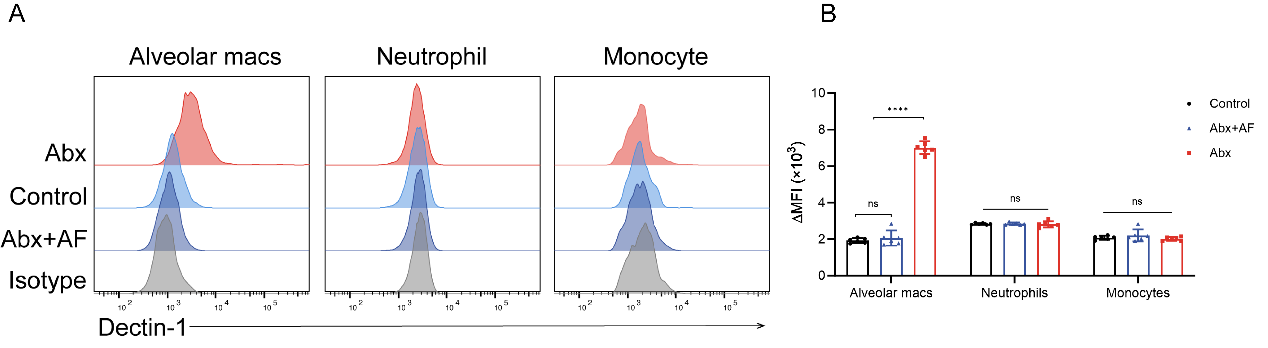


Figure S3. Dectin-1 expression is upregulated in AMs of Abx mice. A, B) Representative flow cytometry histograms of surface molecules on AMs, neutrophils, and monocytes. **** *P*< 0.0001

| 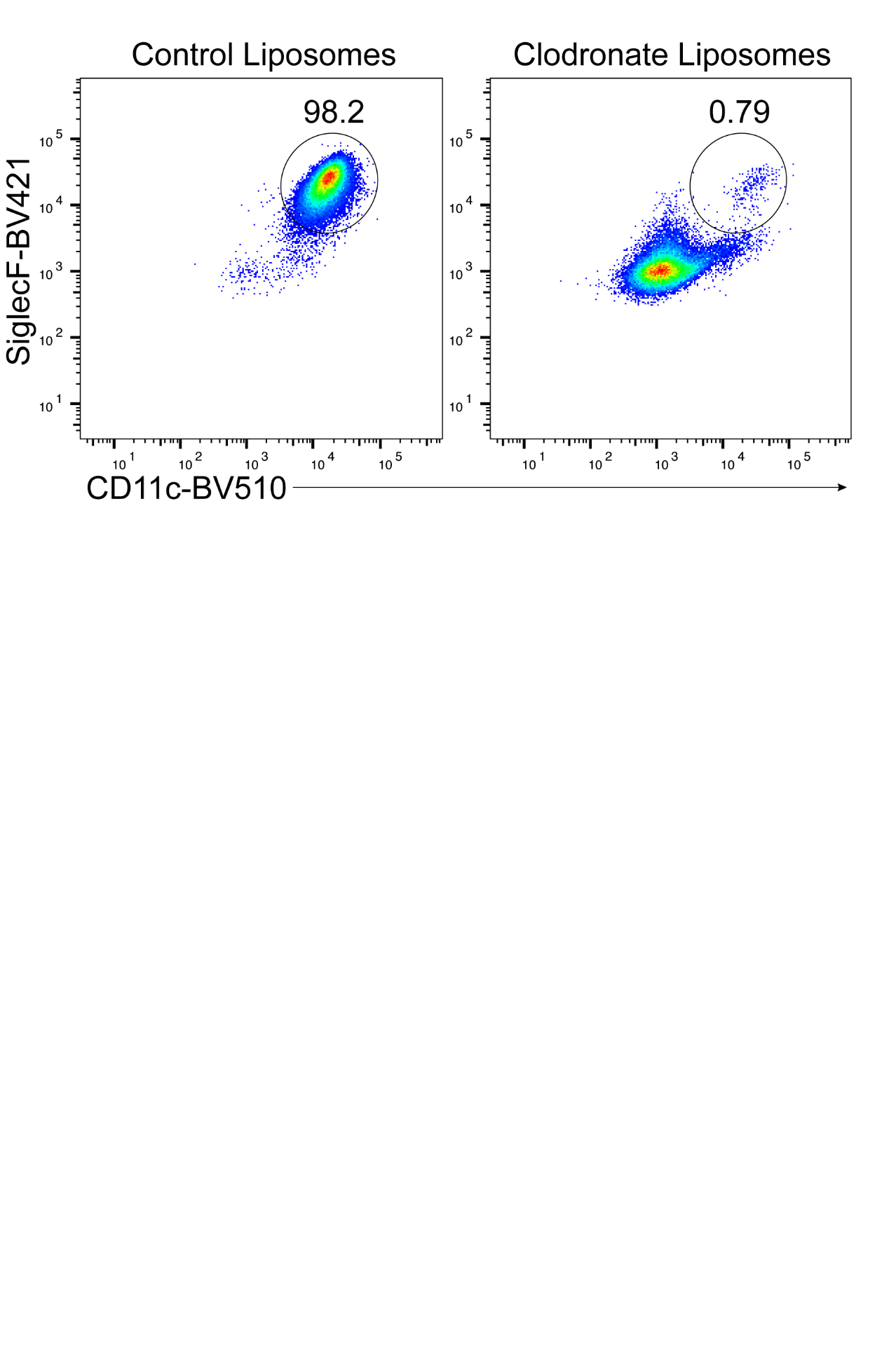 |
| --- |
| Figure S4. The depletion of AMs in mice was successfully achieved using clodronate liposomes. |
| 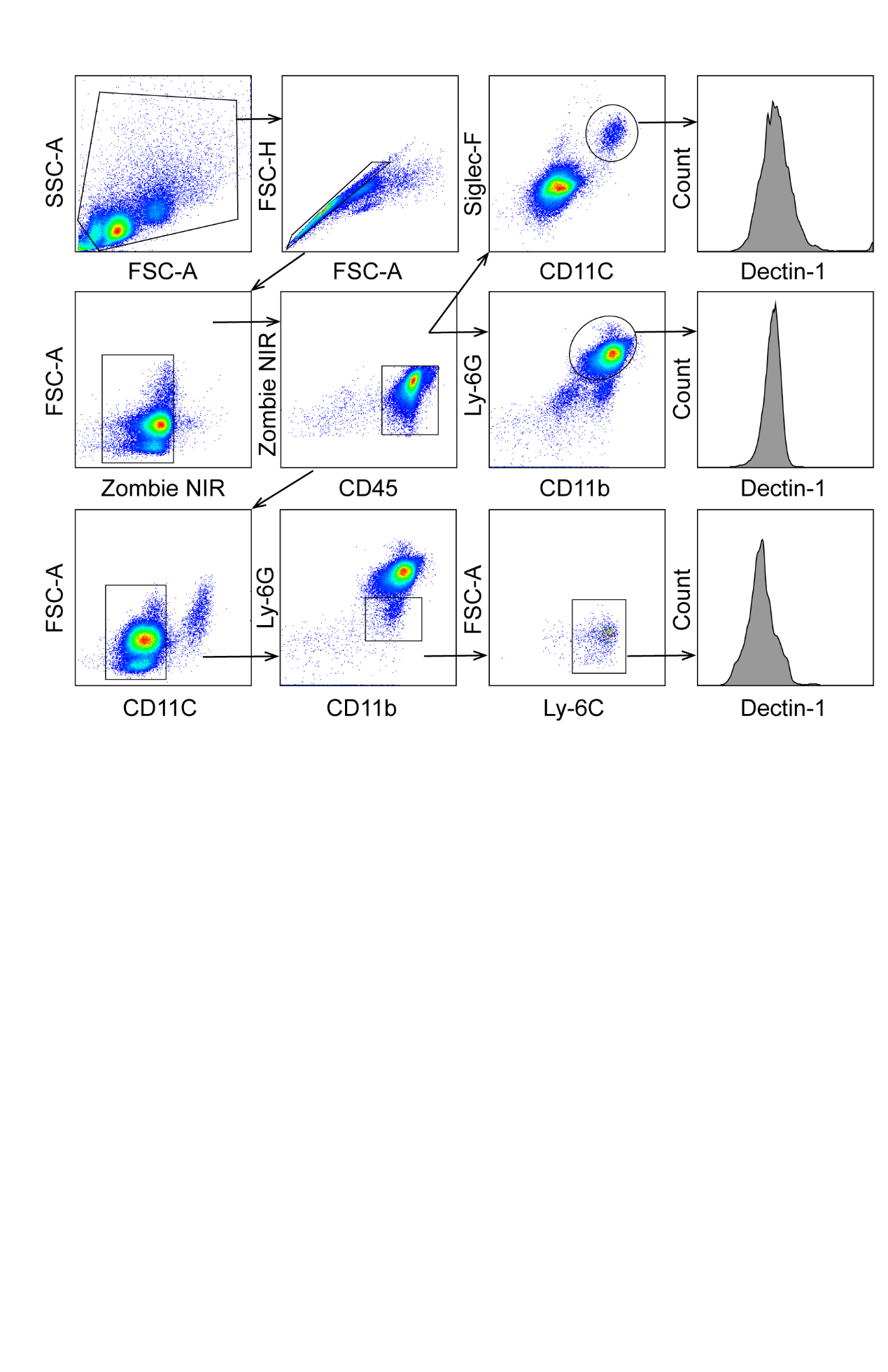 |
| Figure S5. Representative flow cytometric dot plots showing Dectin-1 expression on gated CD11c^+^Siglec-F^+^ alveolar macrophages, CD11b^+^Ly-6G^+^ neutrophils, and CD11c^-^CD11b^+^Ly-6G^-^Ly-6C^+^ monocytes in the lung at 24 hours post *Klebsiella pneumoniae* infection. |

**Reference**

1. Schingnitz U, Hartmann K, Macmanus CF et al. Signaling through the A2B adenosine receptor dampens endotoxin-induced acute lung injury. *J Immunol*. 2010;**184**:5271-9 <https://doi.org/10.4049/jimmunol.0903035>

2. Kajino-Sakamoto R, Fujishita T, Taketo MM et al. Synthetic lethality between MyD88 loss and mutations in Wnt/β-catenin pathway in intestinal tumor epithelial cells. *Oncogene*. 2021;**40**:408-20 <https://doi.org/10.1038/s41388-020-01541-3>
